# Physical or Psychological Therapy? Cognitive Behavioral Therapy or Acupuncture for Subsyndromal Depression among Methamphetamine Users

**Published:** 2019-03

**Authors:** Bijan PIRNIA, Kambiz PIRNIA, Khadijeh BAZYARI, Fatemeh ASLANI, Parastoo MALEKANMEHR

**Affiliations:** 1. Department of Psychology, Faculty of Humanities, University of Science and Culture, Tehran, Iran; 2. Behavioral Sciences Research Center, Shahid Beheshti University of Medical Sciences, Tehran, Iran; 3. Bijan Center for Substance Abuse Treatment, Tehran, Iran; 4. Department of Psychology, Ahvaz Branch, Islamic Azad University, Ahvaz, Iran; 5. Department of Psychology, Karaj Branch, Islamic Azad University, Karaj, Iran; 6. Department of Psychology, Hamedan Branch, Islamic Azad University, Hamedan, Iran

## Dear Editor-in-Chief

Using Methamphetamine is comorbid lifetime anxiety disorders. Sub-syndromal Depression Disorder (SSD) refers to the existence of major clinical symptoms that these symptoms in terms of intensity are not enough for the diagnosis of major depression and does not meet all the diagnostic criteria (1).

Acupuncture is a therapy method used around the world for two thousand years. National Acupuncture Detoxification Association (NADA) is an applied Manuel Protocol of Ear Acupuncture (EA) that is an evidence-based approach.

On the other hand, cognitive-behavioral therapy (CBT) has been associated with promising results in the treatment of substance abuse. Two studies compared the effectiveness of CBT and acupuncture in reducing symptoms of depression. There is not a significant difference between the effectiveness of these two therapies on the depression index in patients with SSD (1). In the study of comparing the effectiveness of EA and CBT on depression in patients with insomnia were not reported significant (2).

According to the conflicting evidence on the effectiveness of the two treatments and aimed to develop effective treatment strategies, this study aimed to compare the effectiveness of two approaches of Ear acupuncture and cognitive therapy on symptoms of depression in patients with SSD. This was a one-site controlled clinical trial conducted in the Bijan Center for Substance Abuse Treatment between Dec 2015 and Jun 2016. Overall, 36 male participants were chosen through Respondent-driven sampling (RDS) were assigned into three groups of CBT, EA and control by Excel software. Subjects were under preliminary assessment for six weeks in a baseline and then six weeks of treatment was conducted on two groups of treatment and the control group received no treatment.

CBT was presented based on the theory of ABC (activating events-belief consequence) that includes six weekly sessions of 1.5–2 h. Intervention was performed by a therapist with a clinical certificate in psychology trained in this approach (first author, B. P). Groups were including 6 to 10 subjects.

**Fig. 1:**
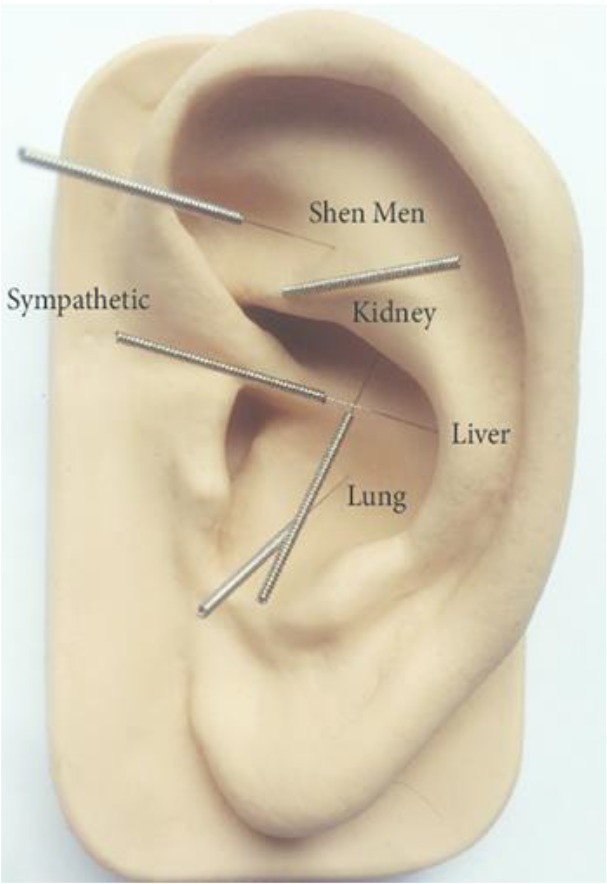
Acupuncture points according to the NADA protocol

EA was performed three times a week for six weeks (eighteen sessions) and the duration of each session was 30–45 min. During the session, five points of ear supposed to be the best points in substance-abusing patients were intervened (Fig. 1). EA was performed in both ears using disposable stainless steel needles (0.25+13 mm) with a depth of 2–3 mm and using manual stimulation method by a trained doctor and an acupuncturist who had a degree and five-years history of the treatment (second author, K. P).

Inclusion criteria were included: 1) 18–40 yr of age; 2) presence of ≥1 but <5 symptoms required for the diagnosis of major depression (MD) based on DSM 4; 3) the symptoms last at least two weeks; 4) informed consent. Exclusion criteria were included: 1) a history of psychiatric disorders or brain injury; 2) unstable medical conditions; 4) participating in any clinical trial in the last four weeks, and 5) allergy to nickel or ear tissue damage. Beck Depression Inventory (BDI) and The Structured Clinical Interview for DSM-4 (SCID-4) were used to detect symptoms of depression below the threshold. According to the non-normal distribution scores of depression, to analyze the data during 12 wk of evaluation, semi-parametric test of GEE (3) through IBM SPSS Statistics Version 20 **(**IBM Corp**.,** Armonk**,** NY, USA**)** was used. Statistical significance was accepted at the level of *P*<0.05.

The informed consent was obtained and the whole process was carried out based on the latest version of the Declaration of Helsinki.

Decrease the craving in the EA group is significant. The mean of (95% of confidence) methamphetamine craving tests was 8.11 (5.91–10.32) in EA group, 9.43 (4.91–13.95) in CBT group and 9.51 (5.18–13.84) in control group (*P*=0.037). Although no significant reduction in depression index in the EA group was observed (*P*>0.05). These findings are consistent with studies (4) that showed that no evidence for the effectiveness of EA in reducing anxiety in drug abusers was observed.

Secondary outcome represents a significant improvement in depressive symptoms in the CBT group (*P*<0.05). Cognitive and behavioral interventions based on the belief that knowledge has a key role in etiology and maintenance of depressive disorders is effective. ABC theory was used in this study. This model can be effective on the symptoms of depression and these findings are consistent with previous studies (1). According to our findings on the effectiveness of acupuncture on craving and effectiveness of CBT on depression symptoms, combining acupuncture treatment and CBT can draw a promising outlook in the treatment of addiction.
